# Effectiveness of Adjunct Robotic Therapy With a Patient-Guided Suspension System for Stroke Rehabilitation Using a 7-Days-a-Week Model of Care: A Comparison With Conventional Rehabilitation

**DOI:** 10.1016/j.arrct.2021.100144

**Published:** 2021-07-22

**Authors:** San San Tay, Christine Alejandro Visperas, Abbas Bin Zainul Abideen, Mark Min Jian Tan, Ei Mon Zaw, Hsuan Lai, Edmund Jin Rui Neo

**Affiliations:** a Department of Rehabilitation Medicine, Changi General Hospital, Singapore; b Department of General Medicine, Changi General Hospital, Singapore; c Rehabilitation Medicine, SingHealth Residency, Singapore

**Keywords:** Gait, Stroke, Rehabilitation, Robotics, BBS, Berg Balance Scale, FAC, functional ambulation category, IQR, interquartile range, LOS, length of stay, MMT, manual muscle testing, mRS, modified Rankin Scale, NIHSS, National Institutes of Health Stroke Scale

## Abstract

**Objective:**

To determine and compare the effectiveness of robotic therapy with a patient-guided suspension system for stroke rehabilitation using a 7-days-a-week model of care with that of conventional rehabilitation.

**Design:**

Retrospective cohort study.

**Setting:**

Inpatient rehabilitation unit of an acute general hospital.

**Participants:**

A total of 100 consecutive patients with stroke (N=100) admitted within a 7-month period who fulfilled the criteria to undergo robotic therapy with a patient-guided suspension system were enrolled in this study.

**Interventions:**

Patients either underwent robotic therapy in addition to conventional therapy (robotic group) or conventional therapy only (control group). There were 50 patients in each cohort.

**Main Outcome Measures:**

FIM and its derivatives (FIM gain and FIM efficiency); Berg Balance Scale (BBS), functional ambulation category (FAC); modified Rankin Scale (mRS); and National Institutes of Health Stroke Scale.

**Results:**

The average FIM gains in both groups were statistically significant (*P*<.01). The robotic group had greater improvement in FAC scores (1.24 vs 0.78, *P*=.007). However, other measurements such as FIM efficiency, BBS, and mRS were not significantly different between the 2 groups. The robotics group reported high patient satisfaction rates, with most patients finding the intervention both beneficial and desirable.

**Conclusions:**

Adjunct robotic therapy has the potential to increase the efficacy of stroke rehabilitation. However, further studies are needed to strengthen the evidence.

Several studies have shown that greater intensity of stroke rehabilitation leads to better outcomes.^1^, ^2^, ^3^ A previous prospective study found a positive relationship between lower limb exercise dose (mean daily number of exercise repetitions) and improved walking speed.^4^ However, increasing the dose of exercise that therapists can deliver is often difficult because of administrative tasks inherent to therapy sessions, such as documentation.^5^ Our unit, like many others, is constantly challenged to provide 3 hours of intense therapy per day in accordance with stroke rehabilitation guidelines.^6^^,^^7^ We considered 7-days-a-week rehabilitation as a method of increasing our exercise dosage.^8^ This was constrained by manpower resources. Robotic therapy enabled us to provide adjunct therapy on weekends, when patients typically do not have regular access to rehabilitation.

Our rehabilitation unit had an opportunity to trial a patient-guided partial body weight–supported suspension system robotic device, Andago.^a^ We conducted feasibility work on this robotic device for a month in 2017. This robotic device is categorized as a patient-guided suspension system.^9^ Although it provides only partial weight support, it allows for over-the-ground walking at a patient-selected speed. Patients who had completed robotic training with a tethered exoskeletal robotic device were allowed to train with Andago. This device was suitable for patients with functional ambulation category (FAC) 1 or 2.

Within the trial month, 17 patients showed significant improvement in function and ambulation, and the service was deemed feasible. The device was subsequently acquired, and a new service was implemented in our rehabilitation unit for adjunct therapy in patients with stroke. This article reports the findings of a service evaluation performed 4 months after the service was started in 2019. The study aims were (1) to determine whether the robotic and conventional therapy groups have comparable demographic and clinical characteristics; (2) to compare the outcomes and determinants of rehabilitation efficiency (namely, FIM efficiency) in both groups; and (3) to describe the patient experience and level of satisfaction in the robotic group.

## Methods

Our acute inpatient rehabilitation unit is part of an acute general hospital. Admissions are of varied diagnoses, but stroke accounts for approximately 40% of the 1200 admissions per year. The stroke rehabilitation consultation service is available Monday through Saturday as well as on public holidays. Generally, most patients are transferred to the acute inpatient rehabilitation unit as early as the second or third day of stroke (median interval of 0 days from stroke unit referral to transfer to inpatient rehabilitation).

This is a retrospective cohort study. A total of 282 electronic medical records were reviewed for patients diagnosed as having stroke who were discharged from our acute inpatient rehabilitation unit between March and October 2019. The study included 100 patients who had a manual muscle testing (MMT) score of at least 3 (antigravity on the Medical Research Council scale), were walking with minimal to moderate assistance (FAC 1-2) on transfer to the rehabilitation unit, and met manufacturer guidelines.

Suitability criteria for robotic therapy in the manufacturer's guidelines included the following: (1) stable vital signs; (2) weight <135 kg and height <2 m; (3) lower limb MMT ≥3 (antigravity) on hip flexion; (4) nil limitations in the range of motion; (5) full weight-bearing status; (6) cognition allows them to participate in the therapy; and (7) ability to adjust/fit the harness to respective body parts.

Further, patients with severe visual problems, infectious diseases, postural instability, bone instability, severe spasticity and contractures, severe movement disorders, unstable vital signs, pregnancy, and structures such as stoma bags or skin lesions that prevented donning of the harness were considered unsuitable for robotic therapy.

**Fig 1:**
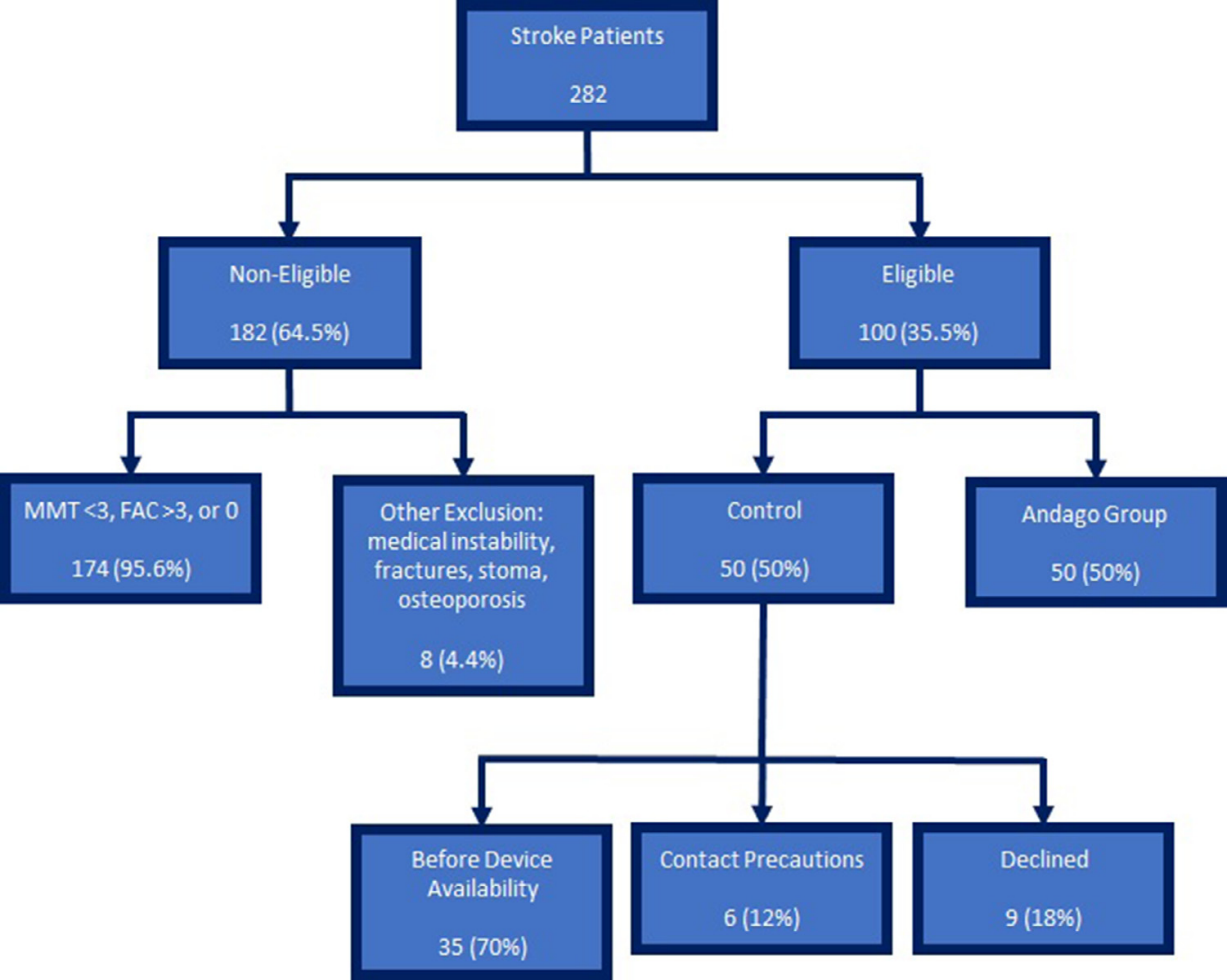
Modified Strengthening the Reporting of Observational Studies in Epidemiology flow diagram showing participant allocation into control and intervention groups as well as reasons for allocation to control group.

Of the 100 eligible patients, 50 consecutive patients who met the stroke criteria, had an MMT ≥3 (antigravity), were walking with minimal to moderate assistance, and fulfilled the manufacturer's criteria but did not undergo robotic therapy formed the control group. Of the 50 control group patients, 35 were admitted before Andago had been operational, and 6 needed to be on contact precautions. One patient declined robotic therapy, and 8 patients were declined robotic therapy by their primary care provider. The first 50 consecutive patients who underwent robotic therapy formed the robotic group (fig 1). Other robotic interventions were not performed in either patient group.

Clinical characteristics such as diagnosis, location of stroke, motor strength, modified Rankin Scale (mRS), National Institutes of Health Stroke Scale (NIHSS), Berg Balance Scale (BBS), FIM, and length of stay (LOS) are routinely documented in our electronic medical records. These data were recorded as a standard of care.

Andago is a relatively new robotic device that is categorized as a patient-guided suspension system. It allows for partial weight support of up to 25 kg on each side. The patient usually starts with weight supports of 2.5-5 kg on each side, which are then adjusted to the minimum weight support required for safe ambulation. The device allows for selection of gait speed by the therapy assistant via a remote control, at a level that is suitable for the patient. Sensors and control algorithms assist the device with propulsion, maneuvering of the frame, and directional adjustments for patient safety. Safety features include collision-sensing autobrakes that activate on minimal impact and sense pauses in the patient's walking. The harness system is another safety measure that prevents the patient from falling. This device allows the patient to explore the environment and motivates the patient because they are usually able to ambulate 200-300 m during the first session.

Conventional therapy included an average of 25 minutes of 1-on-1 physiotherapy, 21 minutes of occupational therapy, and 30 minutes of group therapy on weekdays. On Saturday, patients received an average of 30 minutes of group therapy. Although each session lasted 60 minutes, not every patient attended.

Patients received 4-10 sessions of robotic therapy throughout their stay, with an average of 7±2.34 sessions. The adjunct robotic therapy started when the patients met the criteria for motor power or when their blood pressure was within stipulated limits. The intervention was stopped when the patients were discharged or transferred to another facility. Gait training with Andago was administered on consecutive days, including weekends and public holidays, with each session lasting 30 minutes. During the 30 minutes, each patient underwent gait training and was allowed to ambulate as much as his endurance allowed. The distance that the patient could ambulate was only limited by the patient's endurance and precautions such as blood pressure limits. The device captures the distance ambulated. Approximately 80% of the robotic group received therapy for 7 days a week. The medical records were reviewed after the service was used for 4 months.

Our primary outcome measure was the FIM. FIM and its derivatives, namely FIM gain (difference between discharge and admission FIM) and FIM efficiency, were analyzed. FIM efficiency measures the rate of daily improvement in the FIM score during the rehabilitation stay. The secondary outcomes were pre- and postintervention BBS, FAC, mRS, ambulation distance, and patient satisfaction.

Descriptive statistics were used for age, sex, and stroke diagnosis classifications. Either the unpaired *t* test or the chi-square test was applied to compare the robotic and control groups for continuous and categorical variables, respectively. Nonnormal data were analyzed using the Mann-Whitney *U* test. For postimplementation results, we used both the unpaired *t* test and the Mann-Whitney *U* test to compare changes between the robotic and control groups, whereas paired pre- and postintervention findings were analyzed with the paired *t* test and the Wilcoxon signed-rank test. Data analysis was performed using IBM SPSS version 25.0^b^ and counterchecked independently. All statistical tests were 2-tailed, and a *P* value <.05 was considered statistically significant. For both raw data and derived results, missing data were excluded from analysis.

The centralized institutional review board of our institution (Ref: 2019/2989) reviewed this study and determined that it did not require further ethical deliberation because it was a service evaluation using deidentified data. The study was conducted in compliance with all applicable institutional policies, regulations, guidelines, and study protocols. Informed consent was not necessary because of the nature of the study.

## Results

### General demographics

The demographics of both the robotic and control groups—age, sex, type, and location of strokes—were similar, with no significant intergroup differences. Likewise, there were no significant intergroup differences in the baseline scores for FIM, FAC, mRS, BBS, and NIHSS at admission (table 1).

**Table 1:** Baseline demographics all patients (N=100)

Variables	Control Group (n=50)	Robotic Group (n=50)	*P* Value
**Demographic information**			
Age (y), mean ± SD	67.8±10.28	65.6±10.28	.287 (unpaired *t*)
Sex, n (%)			
Female	14 (28)	15 (30)	.826 (χ^2^)
**Lesion characteristics**			
Stroke type, n (%)			>.99 (χ^2^)
Ischemic	45 (90)	45 (90)	
TACI	2 (4)	2 (4)	
PACI	16 (32)	16 (32)	
POCI	19 (38)	12 (24)	
LACI	8 (16)	15 (30)	
Hemorrhagic	5 (10)	5 (10)	
Stroke site, n (%)			.934 (χ^2^)
Left	25 (50)	25 (50)	
Right	21 (42)	20 (40)	
Bilateral	4 (8)	5 (10)	
**Baseline scores**			
BBS, mean ± SD	19.70±14.19	17.33±13.40	.406 (unpaired *t*)
FAC, mean ± SD	1.78±0.76	1.76±0.80	.898 (unpaired *t*)
mRS, median (IQR)	4 (4-4)	4 (4-4)	.868 (Mann-Whitney *U*)
NIHSS, median (IQR)	5 (3-7)	5 (4-6)	.920 (Mann-Whitney *U*)
FIM, mean ± SD	70.72±20.14	74.64±16.15	.286 (unpaired *t*)

Abbreviations: LACI, lacunar infarct; PACI, partial anterior circulation infarct; POCI, posterior circulation infarct; TACI, total anterior circulation infarct.

### Functional outcomes

The mean BBS of the control and robotic groups at admission was 19.70±14.19 and 17.33±13.40, respectively, indicating that both groups had a high risk of falls and slow walking speed at admission. However, it significantly improved in both groups to 37.38±16.07 and 37.7±14.52, respectively (table 2). The improvements in both groups were significant enough to meet the criteria for minimal detectable change, but neither group improved significantly more than the other (*P*=.244).

**Table 2:** Effect of robotic vs control group on outcomes in all patients

Variables	Control Group		Robotic Group		Effect
**Balance and mobility outcomes**					
BBS, mean ± SD					
Score on admission	19.70±14.19		17.33±13.40		
Score at discharge	37.38±16.07		37.65±14.52		
		*P*<.001 (paired *t*)		*P*<.001 (paired *t*)	
BBS gain, mean ± SD	16.09±7.96		19.65±12.15		*P*=.244 (unpaired *t*)
FAC, mean ± SD					
Score on admission	1.78±0.76		1.76±0.80		
Score at discharge	2.56±0.95		3.00±1.01		
		*P*<.001 (paired *t*)		*P*<.001 (paired *t*)	
FAC gain, mean ± SD	0.78±0.76		1.24±0.89		*P*=.007 (unpaired *t*)
**Disability outcomes**					
mRS, median (IQR)					
Score on admission	4 (4-4)		4 (4-4)		
Score at discharge	4 (2-4)		3 (2-4)		
		*P*<.001 (Wilcoxon)		*P*<.001 (Wilcoxon)	
mRS gain, median (IQR)	−1 (−1 to 0)		−1 (−2 to 0)		*P*=.232 (Mann-Whitney *U*)
NIHSS, median (IQR)					
Score on admission	5 (3-7)		5 (4-6)		
Score at discharge	2 (1-4)		3 (1-4)		
		*P*<.001 (Wilcoxon)		*P*<.001 (Wilcoxon)	
NIHSS gain, median (IQR)	−3 (−4 to −1)		−2 (−4 to 0)		*P*=.355 (Mann-Whitney *U*)
FIM, mean ± SD					
Total score on admission	70.72±20.14		74.64±16.15		
Total score at discharge	82.64±21.33		87.96±17.56		
		*P*<.001 (paired *t*)		*P*<.001 (paired *t*)	
FIM gain, mean ± SD	11.92±10.92		13.32±11.04		*P*=.525 (unpaired *t*)
**Quality outcomes**					
Total LOS, median (IQR)	18 (12-21)		16 (12-18)		*P*=.125 (Mann-Whitney *U*)
Rehab LOS, median (IQR)	12 (9-17)		12.5 (8-15)		*P*=.266 (Mann-Whitney *U*)
mean ± SD	14.50±9.40		11.96±4.59		
FIM efficiency, median (IQR)	0.86 (0.27-1.86)		1.29 (0.71-2.29)		*P*=.162 (Mann-Whitney *U*)

The improvement in FAC was greater in the robotic group than in the control group, with a mean gain of 1.24±0.89 vs 0.78±0.76. This was statistically significant (*P*=.007). The mean FAC for the robotic group had changed from “continuous support for mobility” to “standby-help-or-verbal-supervision for mobility.”

All patients experienced improvements in their disability outcomes on discharge, namely the mRS (*P*<.001) and NIHSS (*P*<.001), when compared with admission. In the robotic group, the median mRS improved by 1 grade, from 4 (interquartile range [IQR], 4-4] at admission to 3 (IQR, 2-4) at discharge. In the control group, the median mRS remained at 4 at both admission and discharge, although the mRS range improved (IQR, 4-4 to IQR, 2-4) on discharge. However, no significant difference was found in the improvement in mRS scores between the control and robotic groups (*P*=.232). The median NIHSS scores decreased from 5 (IQR, 3-7) to 2 (IQR, 1-4) in the control group, whereas in the robotic group it decreased from 5 (IQR, 4-6) to 3 (IQR, 1-4). However, there was no significant difference in the NIHSS scores between the 2 groups (*P*=.355).

The average FIM score in both groups was within the moderate stroke category at admission. The average FIM gains within each group were statistically significant. The FIM gain in the robotic group was 1.4 points higher, but it did not reach statistical significance (*P*=.525).

The median LOS in hospital was 16 days (IQR, 12-18d) for the robotic group and 18 days (IQR, 12-21d) for the control group (*P*=.125). However, the median LOS in the rehabilitation unit was 12.5 days (IQR, 8-15d) days for the robotic group and 12 days (IQR, 9-17d) for the control group (*P*=.266). The median FIM efficiency was 1.29 (IQR, 0.71-2.29) in the robotic group and 0.86 (IQR, 0.27-1.86) in the control group (*P*=.162). The average distance ambulated per session on the Andago was 358 m.

### Patient satisfaction

Most patients tolerated robotic therapy well and were either satisfied or very satisfied with it. Most preferred robotic therapy as an adjunct to conventional therapy rather than conventional therapy alone. They would even recommend it to other patients and are likely to use it again, if needed (table 3).

**Table 3:** Survey questions and tabulation of responses given by the robotic group

Response Category	Responses, n (%)	Response Category	Responses, n (%)
How satisfied are you with the robotic therapy?		How would you compare undergoing a combination of robotic and conventional therapies vs undergoing conventional therapy alone?	
Very satisfied	28 (56)		
Somewhat satisfied	6 (12)		
Neither satisfied nor dissatisfied	3 (6)	Much better	17 (54)
Somewhat dissatisfied	2 (4)	Somewhat better	12 (24)
Very dissatisfied	0 (0)	About the same	8 (16)
Unable	1 (2)	Somewhat worse	1 (2)
Blank	10 (20)	Much worse	0 (0)
		Don't know	1 (2)
To what extent was the robotic therapy beneficial to your disability?		Unable	1 (2)
		Blank	10 (20)
Very helpful	27 (54)		
Somewhat helpful	10 (20)	Would you recommend the robotic therapy to other patients? (Scale of 0-10, not likely to extremely likely)	
Neither helpful nor unhelpful	2 (4)		
Somewhat unhelpful	0 (0)	10/10	14 (28)
Very unhelpful	0 (0)	9/10	4 (8)
Unable	1 (2)	8/10	11 (22)
Blank	10 (20)	7/10	1 (2)
		6/10	1 (2)
Which of the following words would you use to describe robotics? (You may choose more than one word)		5/10	5 (10)
		4/10	1 (2)
Useful	29	3/10	0 (0)
Reliable	16	2/10	0 (0)
High quality	16	1/10	0 (0)
Unique	7	0/10	0 (0)
Ineffective	2	Unable	3 (6)
Unreliable	1	Blank	10 (20)
How likely are you to undergo the robotic therapy again, if required?			
Extremely likely	13 (26)		
Very likely	21 (42)		
Somewhat likely	3 (6)		
Not so likely	2 (4)		
Not at all likely	0 (0)		
Unable	1 (2)		
Blank	10 (20)		

### Adverse events

The medical team assessed the patients’ suitability to continue robotic therapy on a daily basis. There were no adverse events, such as injuries or pain, that caused disruption of treatment. There were no reports of discomfort, pain, or injury that surfaced through the patient satisfaction survey.

## Discussion

### Patient-related outcomes

FIM was our primary outcome measure. The motor FIM efficiency and total FIM efficiency in the robotic group was higher than those of the control group, but these did not reach statistical significance. Greater improvements in BBS (a reliable scale in identifying people with increased fall risk that can be applied to patients with stroke)^10^^,^^11^ and a bigger shift in the IQR for mRS scores (measuring the degree of disability or dependence in the daily activities) were observed in the robotic group. However, these did not reach statistical significance.

A statistically significant difference was observed in FAC improvements between the robotic and control groups. Robotic therapy increases the number of gait cycles. This may further concur with previous findings that more repetitions result in better gait.^4^ Conventional physiotherapy involves stretching, strengthening, balance training, and gait training. In contrast, the robotic therapy was solely dedicated to gait training. In an observational study of inpatient physical therapy, it had been observed that patients performed a variety of tasks with limited focus on any one task.^12^ Robotic gait training also enables an exceedingly high number of gait cycle repetitions. The usual maximum distance that a patient ambulated with the physiotherapist in conventional therapy was approximately 60 m in the inpatient environment, whereas the distance ambulated per patient per session on the robotic device was 358 m on average. This may have made a difference in the improvement observed in the FAC scores, which was the only statistically significant outcome. Achieving an FAC of 3 also meant that more patients would only need supervision or even less assistance for mobility at discharge, which has implications in reducing the burden of care. Newer studies show that high-intensity stepping training during inpatient stroke rehabilitation improves outcomes.^13^ Specificity, amount, and intensity of locomotor training are other important factors.^14^^,^^15^ However, because these are not routinely measured in our unit, we used the distance ambulated as a surrogate for both the number of steps taken and an indirect indicator of endurance.

Most patients had very positive experiences with robotic treatment, as evidenced by the postintervention survey outcomes. Patients also felt comfortable and confident when robotic therapy was applied. Another study reported that the intervention is safe and that most patients enjoyed the experience.^11^

In this study, patients benefitted from the 7-days-a-week therapy. They would have spent 2-3 weekends in hospital for an average stay of 12-16 days because conventional therapy was not available on Sundays. In our unit, most patients with stroke were seen by a rostered physiotherapist on Saturdays. Some patients may decondition and experience reversal in their therapy gains over the weekend because of the lack of activity. Robotic therapy, which can be conducted by a therapy assistant, ensures that the patients remain active even on weekends. The days saved could be because of the “compressed” temporal context of having 7-days-a-week therapy.

### Study limitations

The limitations of this study, other than the restricted sample size, included the lack of longitudinal follow-up at 3 months or longer. Unlike other studies wherein patients received either conventional or robotic therapy, one may argue that there may be other confounding factors in this study. However, this study was primarily conducted as a service evaluation of using robotic therapy to complement conventional therapy rather than a head-to-head comparison. The significant improvement in FAC may be not solely because of the robotic device. It may also be attributed to the increased exercise dose, as evidenced by the duration in therapy as well as the distance ambulated.

Our preliminary findings suggest that robotic therapy, when used as an adjunct in a 7-days-a-week setting, has the potential to increase efficiency in rehabilitation. It may also boost the patients’ confidence, enabling them to return to community life earlier.

Other than FAC, results of the robotic and control groups, although favorable, did not achieve statistical significance in terms of FIM, BBS, mRS, and LOS. This could be attributed to the relatively small number of patients in each cohort. Another explanation is that the rehabilitation LOS is generally short because of the model of care in our unit. Therefore, it would be challenging to demonstrate a greater reduction in the LOS with this sample size. Robotic therapy for suitable patients with stroke using a patient-guided suspension system could be implemented on a broader scale. This should also be considered for patients with other rehabilitation diagnoses and in other settings, such as in the geriatrics ward, to prevent deconditioning. Further studies should be performed in other diagnostic groups.

## Conclusions

A 7-days-a-week model had the potential to increase rehabilitation efficiency and improve the FAC scores in our study using a patient-guided suspension system robotic device for selected groups of patients with stroke with FAC 1-2. However, further studies are needed to strengthen the evidence.

## Suppliers

a. Andago; Hocoma.
b. IBM SPSS, version 25.0; IBM Corp.
